# Report from the Medical Wards of the Illinois General Hospital

**Published:** 1853-11

**Authors:** N. S. Davis

**Affiliations:** One of the Physicians to the Hospital, and Prof. of Pathology, Practice of Medicine, and Clinical Medicine in Rush Medical College


### THE NORTH-WESTERN MEDICAL AND SURGICAL JOURNAL.

NEW SERIES.

VOL. II. NOVEMBER, 1853. NO. 7.

### ORIGINAL COMMUNICATIONS.

ART. I.—*Report from the Medical Wards of the Illinois General Hospital*

By N. S. DAVIS, M. D., one of the Physicians to the Hospital, and Prof. of Pathology, Practice of Medicine, and Clinical Medicine in Rush Medical College.

(*Continued from last Number.*)

DELIRIUM TREMENS.—In the first part of this report it was stated that six cases of Delirium Tremens had been admitted into the Medical Wards of the Hospital.

Of these, four were simple uncomplicated cases, commencing from one to three days after a protracted period of intoxication.—All these were readily controlled by full doses of morphine, repeated at such intervals as to preserve uniformity of effect, and recovery took place in from four to ten days. The other two cases commenced while the patient was still indulging in frequent and excessive alcoholic potations, and were complicated, one with acute inflammation of the mucous membrane of the stomach, the other with dysentery. The latter M. F., a young man, was brought to the Hospital on the 25th June. He had been drinking the same day. His brain and nervous system were highly excited, his mind extremely fearful, and haunted with images of every shape, his skin warm, pulse quick and hard, eyes injected, abdomen tender, evacuations frequent, mucous and small in quantity. Directed entire quiet, with a powder containing one grain of morphine and three of camphor to be given at once, and half of the quantity repeated every hour until he became quiet. Then the same medicine was to be given at longer intervals.

JUNE 26th.—Patient had a little unquiet sleep during the night, but is much excited again this morning—has had no discharges from his bowels—pulse less quick and hard—skin moist; directed tincture of opium 40 drops, with chloroform 10 drops, repeated every 2, 3, or 4 hours, according to the effect in preserving quiet.

JUNE 27th.—Patient slept considerable during the night, appears more rational this morning; skin cool—pulse less frequent but firm. No evacuation of bowels since first day of admission.—Directed a laxative, consisting of one table spoonful of castor oil, with 30 drops of oil of turpentine, to be followed immediately by the tincture of opium and chloroform sufficient to induce sleep.

JUNE 28th.—The oil operated freely, and he appears quite free from excitement this morning; the skin is cool—the pulse 90 and weak—looks pale—tongue moist and clean, and sweat freely during the latter part of the night, but slept very little. Directed him to take a powder of sulph. morph. one grain, and sulph. quinine three grains, every three hours, and some beef tea for nourishment. During the middle of the day, while the nurse was momentarily absent from the room, the patient quickly raised the window and made good his escape. In the evening he entered a shop in a distant part of the city, and acted so unusual that some police officers were called in, which adding to his fears and hallucinations, caused him to make a desperate resistance to their attempts to arrest him. The officers being ignorant of his true condition, subdued him by violence and forcibly carried him to the watch-house, where he suddenly expired, almost immediately after their arrival.

The immediate cause of so sudden and unfortunate a result, was not satisfactorily ascertained. The intense cerebral excitement produced by the forcible arrest doubtless aided materially in producing the mischief.

The remaining case of delirium tremens was admitted into the hospital on the nineteenth of January. He was a Canadian by birth, about 30 years of age, and had been drinking intoxicating liquors excessively and almost daily for the last twelve months.—The first distinct symptoms of delirium, had commenced on the day previous. At present, his face is flushed; skin hot and dry; pulse quick, small and hard; tongue clean, moist, and redder than natural, and unusually pointed at the tip; much thirst; no appetite; frequent vomiting; epigastric region full and tympanitic, and very tender to pressure. His muscular movements were quick and tremulous, and his mind constantly agitated with fearful images and horrid phantoms, not only preventing sleep, but keeping the whole system in a state of strong excitement. Directed the application of a mustard sinapism to the epigastrium, and the exhibition of a powder composed of sulph. morph. $\frac{1}{2}$ gr., and sub. murias hydrag. 3 grs., every *two* hours. After four doses have been taken, give a table spoonful of castor oil with 30 drops of oil of turpentine to move the bowels, following the operation immediately by sulph. morph. $\frac{1}{2}$ gr., and sub. murias hydrag. 1 gr. every hour until sleep is induced.

JAN. 21st.—Mind more tranquil, pulse less frequent and hard, skin more moist, the abdomen less tympanitic but still somewhat tender to pressure, and occasional vomiting, with much thirst and a craving for alcoholic drinks. Directed the powders of morphine and calomel to be continued every three hours, with warm hop fomentations over the abdomen.

JAN. 22nd.—All the symptoms still further improved; mind quite tranquil, but still much epigastric tenderness and disposition to reject all nourishment. Directed a powder composed of sulph. morph. $\frac{1}{3}$ gr., blue mass 2grs., and bi. carb. soda 3 grs., to be given every four hours. Also mustard sinapisms over the abdomen sufficient to keep up a moderate counter-irritation.

JAN. 23d.—Patient still remains quiet, sleeps some, pulse not more than 98 per minute but small, skin moist, and less disposition to vomit. The gums are slightly touched with mercurials, the bowels have not been moved during the last 48 hours and are more distended. Directed the powders to be discontinued and a laxative given, consisting of castor oil $\frac{1}{2}$ oz., and ol. terebinth $\frac{1}{2}$ dr., with a weak solution of alum to wash the mouth.

JAN. 24th.—The oil given yesterday was slow in operating, and the patient passed wholly beyond the influence of the morphine previously taken, and soon began to exhibit symptoms of great nervous agitation and excitement, accompanied by a quicker and more feeble pulse, and greater prostration. Directed sulph. morph. $\frac{1}{2}$ gr. every one, two, or three hours, as might be necessary to produce and perpetuate a more tranquil state of the nervous system. Warm fomentations were again applied over the abdomen, and chicken tea allowed in small quantities.

JAN. 25th.—Less excited and tremulous; mind less disturbed; the epigastric region very tender to pressure, and the bowels moving every two or three hours, the discharge being thin, serous and of a reddish color. The pulse continues small and quick; the tongue red and pointed; and occasional vomiting. Directed the application of a blister to the epigastrium, and the following emulsion internally, viz: Oil of Turpentine ʒij.

Tinct. Opii, ʒij.

Gum Arabic }

White Sugar } aa ʒiij.

Water, ℥ij., mix very thoroughly and give one tea-spoonful every two hours, with sulphate of morphine $\frac{1}{4}$ gr. in each dose.

JAN. 25th.—Mind and nervous system more tranquil, and less inclination to reject food or drink, though the bowels continue to move often. Continue same treatment, with two or three spoonfuls of beef-tea every three hours.

JAN. 27th.—Symptoms of delirium tremens all subsided, but the patient much prostrated, with a highly irritable state of the gastric and intestinal mucous membrane. Directed the same medicine internally once in *three* hours, and between each dose of the beef-tea give two table spoonfuls of sweet milk mixed with lime-water, in the proportion of three of the former to one of the latter.

JAN. 29th.—Patient has continued slowly to improve, the stomach and bowels less irritable, and abdomen less tender to pressure. Continue the emulsion once in 4 hours with the morphine *only* at night.

JAN. 30th.—Patient remains quiet; abdomen less tender; pulse less quick; no alvine evacuations, but the urine is scanty and irritating to the urethra, and there remains a sense of burning near the cardiac orifice of the stomach increased by food or drink with entire loss of appetite. Discontinued the emulsion and morphine, and directed an infusion of juniper berries and uva ursi, to be given in doses of a wine-glass-full every three hours, and the same quantity of a mixture of milk 3 parts and lime-water 1 part, between each dose of the infusion—also a small blister over the cardiac region of the stomach.

FEB. 2.—The patient continued slowly to improve until last evening, when a copious diarrhœa returned with vomiting, occasioning rapid prostration. This morning his skin is covered with a cool perspiration; eyes sunken; pulse small and feeble; tongue moist and clean; and extremities cold. Directed for him a mixture of equal parts of brandy, milk and water, a wine-glassful to be given every hour. And also, a tea-spoonful of the emulsion of turpentine and laudanum every 4 hours.

FEB. 4th.—All the symptoms improved, though the discharges continue frequent and of a dyesnteric character. Ordered that the emulsion should be continued every 4 hours, and between each dose a powder composed of pulv. opii. 1gr., acetate of lead 2 grs., and diminish the punch one half.

FEB. 6th.—All the symptoms still further improved, except the irritation of the colon and rectum which continues to give rise to frequent reddish serous discharges. Discontinued all other remedies, and gave the patient pulv. opii. 1 gr., and chloride of sodium 15 grs. every three hours, and boiled milk for nourishment. This treatment was continued, only diminishing from time to time the quantity and frequency of the doses through six or eight successive days. During that time the irritation of the bowels disappeared, the strength and appetite returned slowly, and after a protracted convalescence his health was fully restored.

The foregoing was a severe example of that most dangerous class of cases, in which the delirium is complicated with gastro-enteritis, although the symptoms of the latter are often masked almost completely by those of the former. In this case the patient made no complaint of pain or bad feeling in the stomach on admission, and yet the red and pointed tongue, the small, quick, and hard pulse, the tenderness of the epigastrium, and the quick rejection of whatever was taken into the stomach as food or drink, sufficiently indicated the nature and importance of the gastric complication.

*Chronic Cases.*—Among the 78 cases of chronic disease treated during the period of time included in this report there are many presenting points of practical interest. But my time and space will permit me to allude to only a few of them.

*Tubercular Phthisis.*—The introduction of cod-liver oil into general practice, and the hopes excited by the favorable reports of many eminent in the profession, renders it desirable to place on record all the facts that will aid in establishing the therapeutic value of the remedy. Ten cases of tubercular phthisis were received and treated in the medical wards under my care. Of these five were admitted in the advanced stage of the disease, with extensive cavities in one or both lungs, and all died in the hospital. One was a man over 50 years of age, who had long been addicted to the excessive use of *intoxicating* drinks. He was much emaciated and extremely feeble. He had a large cavity in the upper lobe of the right lung, indicated by well marked pectoriloquy, cavernous respiration, and great depression or contraction of that infra clavicular region of the chest. He lived only 48 hours after admission. Two other cases out of the ten, were in persons long accustomed to the habitual use of alcoholic beverages. I mention this fact in reference to these well marked cases, because it has some bearing on the doctrine advocated recently, that alcohol is a preventive of tubercular diseases. One of the five fatal cases, was a man aged about 70 years, who had manifested symptoms of phthisis during the last *ten* years. The disease progressed slowly. He was admitted into the hospital about twelve months before his death.

At that time the infra clavicular regions were contracted in their antero-posterior diameter, dull on percussion, with sub-mucous ronchus, prolonged respiratory murmur, and increased vibration of voice. Both the symptoms and physical signs indicated extensive tubercular deposit with softening, but no well marked cavities. Being supported by an association of his countrymen, he was supplied with wine, a nutritious diet, and cod-liver oil, which he took during most of his residence in the ward. The disease, however, steadily progressed, leading to the formation of cavities, and ultimately to symptoms of perforation of the pleura of the left side and death. Cod-liver oil either alone or combined with the acetated tincture of opium and phosphate of lime was given to the other three fatal cases, but without any apparent beneficial effect. Of the remaining five, two presented the disease in its early stage, consisting of tubercular deposits in the upper lobes of both lungs sufficient to produce dulness on percussion, irregular and prolonged respiratory murmur, increased vibration of voice, and visible contraction of the antero-posterior diameter of the chest under the clavicles.

One of these remained in the hospital only one month, during which time she was kept on a plain nutritious diet, with a table spoonful of cod-liver oil and a powder composed of phosphate of lime 10 grs., and phosphate of iron 5 grs. three times a day. Her general health slowly but decidedly improved up to the time of her leaving. She was twenty-one years of age and a native of Ireland.

The other was a man aged 35 years, who was sent to the hospital for the purpose of having one leg amputated on account of a scrofulous inflammation of the knee joint. Finding his lungs extensively tuberculated, the surgeon declined operating on the leg, and the patient was put upon treatment essentially the same as that just stated, alternated occasionally with the use of iodine or its compounds.

He remained in the hospital 4 months, during which time the disease of the knee joint improved slowly, but the tubercular affection of the lungs progressed steadily, giving rise to increasing emaciation, purulent expectoration, &c. In two other cases, the disease was in the early period of the stage of softening, and the patients remained in the hospital too short a time to gain any appreciable effects from treatment. The remaining case was a man aged 35 years, a native of Ireland. On admission he presented the following symptoms, viz: a moderate degree of emaciation; pulse 90 per minute; breathing short; frequent cough with a pretty copious muco-purulent expectoration; appetite but little impaired, and bowels regular. Inspection of the chest showed a well marked depression of the infra-clavicular region of the right side when compared with the left; decided dulness on percussion over the same region; also at a point about two inches below the clavicle, there was pectoriloquy and cavernous respiration, with a sub-mucous ronchus all around it. The left side of the chest gave no morbid sounds except an exaggerated or puerile respiratory murmur. Entertaining no doubt of the existence of tubercular deposits and an abscess limited to the upper part of the right lung, he was directed to take during the first week five grains of iodide of potassa disolved in a *tea-spoonful* of the saturated tincture of cimicifuga racemosa root three times a day, and a mixture of the compound honey of squills (hive syrup) and camphorated tincture of opium, to allay the cough, with milk, good bread, and a little tenderly cooked meat for diet. At the end of that time he began to take the cod-liver oil in doses of one table spoonful three times a day, mixed with 10 drops of acetated tincture of opium. Also an infusion of the Lycopus Virginicus in doses of a wine-glassfull three or four times a day. He occasionally also took the hive syrup and paregoric to allay cough. This treatment was continued until he left the hospital, a period of two months. During the first three weeks the expectoration continued copious and purulent, the emaciation increased, his pulse became quicker in the evening and he suffered from night sweats. This treatment, however, was continued with the addition of one or two doses of phosphate of lime and phosphate of iron daily, and during the last five or six weeks his improvement was uniform and well marked. When he left the hospital he had gained considerably in flesh and strength, cough less frequent; expectoration diminished and less purulent; but the infra-clavicular space on the right side remained depressed and duller than the other, though the sub-mucous ronchus had nearly disappeared, and the cavernous respiration was less perfect. He went into the country and took exercise in the open air, lived on plain but nutritious diet, and took some cod-liver oil, though less regularly than while in the hospital.

Some eight months have now elapsed and he is still enjoying a good degree of flesh and strength, without much cough or expectoration, but I have had no opportunity of examining his chest to ascertain the actual condition of his lungs since he went to the country.

This was doubtless one of those cases in which the tubercular deposit occupied only a limited portion of one lung. Having passed through its several stages there, and the system being well supported, a more or less complete cicatrization took place, giving the appearance of positive recovery.

*Uterine Hemorrhage.*—Mrs. T. aged 30 years, married, naturally of good constitution, was admitted into the hospital, May 25th.

About eight months previous, finding her menstrual functions interrupted, and supposing herself pregnant, she attempted to produce abortion by introducing a wire into the mouth of the uterus to destroy the fœtus. This hazardous experiment was followed immediately by flowing and symptoms of moderate inflammation. The latter soon subsided, but the flowing continued moderately up to the time of admission. Sometimes it was accompanied by pain and the discharge of dark and offensive clots, but generally the discharge of blood was small, fresh in appearance and pretty constant. She was feeble in strength, anemic, and troubled with dull aching pain in her back. She said she had been much of the time under the care of a physician, who had prescribed Acetate of Lead and opium, and a variety of other astringents internally, with the daily use of astringent washes, thrown into the vagina. On examination, per vaginam, the mouth of the uterus was found slightly relaxed and open, the body of it apparently enlarged and the hæmorrhage evidently proceeding from within the cavity. Believing the hæmorrhage to arrive from defective contraction of the uterus, and wishing to remedy this on the one hand, and to counteract the anemic state of the blood on the other, I directed for her the following mixture, viz: ℞. Tinct. Ergot ℥ij.
Tinct. Ferri Murias ℥j.
mix, and give one fluid drachm three times a day in sweetened water.

She was also directed to wash out the vagina once a day, with a female syringe, and a solution of alum containing one drachm of the latter, to half a pint of cold water. She was required to remain at rest in a horizontal posture, and avoid all stimulating articles of diet and drink.

On the third day after the commencement of the treatment, a considerable quantity of dark, offensive, and half dissolved clots of blood passed off, accompanied by some pain. After this the flowing pretty rapidly diminished, and at the end of one week was entirely suppressed. The solution of alum as a local application was discontinued soon after the discharge ceased, but the drops were continued internally ten days longer; during which time, she rapidly gained strength, the red color returned to her lips, and she was allowed to sit up, and during the last few days to walk about freely. She was discharged during the third week, apparently quite well.

*Spinal Irritation.*—Mr. D. B., a native of Ireland, aged about 30 years, was admitted into the Hospital on the 17th of January.

His countenance exhibited that anemic, sallow aspect, so generally seen in connection with chronic intermittents, his pulse was slightly increased in frequency, soft and easily compressed;—tongue clean and natural; bowels slightly costive; skin rather dry and slightly above the natural temperature; the spleen was enlarged, its inferior margin projecting below the ribs, and tender to pressure. The most singular feature of the case was an entire inability to maintain an erect position and a morbid sensitiveness of the whole cutaneous surface. The slightest touch on any part of the surface gave rise to painful sensations and nervous agitation, *slight touches* being more distressing than firm pressure, except over the dorsal vertebræ. Pressure on this latter place, would instantly produce universal muscular agitation and spasms An attempt to assume the upright position or even to raise the head from the pillow, would cause giddiness, rapid shaking of the head and a tremulous motion of the eyes in their sockets. All that could be learned of his history was that he had had intermittent fever most of the time since August. He was ordered the following prescription, viz: ℞ Pulv. Doveri grs. 20.

Sub. Murias Hydrarg grs. 20.

Bi Carb Soda grs. 12, mix, divide into 4

doses, and give one every 4 hours until all are taken, then exhibit a laxative of castor oil.

On the 19th his symptoms had undergone no change except in relation to the bowels and spleen. The former had been freely moved and the latter was reduced in size and less tender. He was then ordered a powder containing chloride of sodium 10 grs., ferrocyanuret of iron 2 grs., three times a day, and a pill containing ext. hyosciamus 1 gr., sulph. ferri, 1gr., blue mass 1 gr., at bed time, with a diet of animal broth. This treatment was continued until the morning of the 22nd., when finding the spleen reduced to the natural size, the bowels regular, and the gums slightly affected with the mercurial; and yet no improvement in the symptoms apparently connected with the spinal irritation. the previous medicines were all omitted, and the following ordered in their place, viz : ℞ Sat. tinct. cimicifuga racemosa root, ℥ij.
Tinct. stramonii seeds. ℥ss.
Iodide of potassa, ʒiss., mix,
give one fluid drachm 4 times a day, and apply a blister over the upper dorsal vertebræ. Continue animal broth for nourishment.

He continued this treatment with a repetition of the blister up to the 29th, with only a very moderate degree of improvement.— Some slight indication of chills being manifested, he was directed to discontinue the drops, and take the following powder 4 times a day, viz : ℞ Chloride sodium, grs. 10.
Sulph. quinine, " 2.
Phosphate of iron, " 5., mixed.

The powders seemed to produce a dull head-ache, and at the end of three days, he was restricted to one powder at bed-time, and the tinctures containing Iodide of Potassa, again given three times a day. This treatment was continued with only slight variation until the tenth of March. Repeated small blisters were also applied to the spine. He improved very slowly, being at the date last named, only able to sit up when helped into a chair, but every attempt to help himself produced almost as much agitation and muscular derangement as at first. Still his appetite was good, and the digestive respiratory, and circulatory functions natural. I now omitted the Iodide of Potassa and Stramonium and continued the Tinct. of Cimicifuga root alone, in doses of a tea-spoonful repeated three times a day, and caused three or four quarts of cold water, to be poured in a gentle stream on his head and upper part of the spine every morning. This seemed to produce a marked and decidedly beneficial effect. He was able in a few days to walk across the room, and was discharged on the 25th of April quite well.

I saw him only a few days since, still enjoying very good health. There is every reason to suppose that an earlier resort to cold douche or showering would have produced a much more speedy recovery.

In making the foregoing report, which I fear has extended to a tedious length, I aimed not merely to give the general results of treatment, as applied to different classes of disease, but have also endeavored to give such a detail of symptoms as would enable the reader to compare the cases treated, with those called by the same name, occurring under his own observation. The want of attention to this on the part of many who have published the results of experience, has very materially lessened the value of the facts communicated. Few diseases are susceptible of greater variation in their *specfic* or individual character, than fevers, dysenteries, pneumonias, &c. Hence to make the results of experience valuable to others, it is not sufficient merely to state the medicines given and the proportion of recoveries, but enough of the actual symptoms must be stated to enable each reader to compare the character of the disease treated with cases of the same class, in his own field of practice. The neglect of this has led to much controversy, and much contrariety of opinion, in relation to the value of medicinal agents in the treatment of particular diseases.

Thus Dr. Fenner, and some others in the south fully entitled to our confidence, insist that large doses of Quinine will cut short an attack of Typhoid fever, and set forth the actual results of treatment for proof. Many others, like myself, find Quinine in any dose from 2grs., up to 20grs., much more likely to aggravate the Typhoid cases occurring in our practice, than to relieve them. Yet we all claim to be treating one and the same disease—*Typhoid Fever*.

But it requires only a moderate degree of attention to the *symptoms* of the cases treated by Dr. Fenner, with Quinine, to perceive a wide difference between them and those which have come directly under my observation. Judging from the symptoms as he has given them in his brief report of cases, all his patients presented, at least in the early stage, a high grade of febrile excitement, characterized by a hot skin, quick pulse, flushed face, head-ache, thirst, activity of the capillary circulation, acuteness of sensibility, &c. In a word, the grade of febrile action was closely analagous to that active irritative excitement which exists in the hot stage of intermittents and remittents. But the typhoid fever as it occurs here almost invariably steals upon its victim slowly. He first feels dull and indisposed to active mental or physical exertion.—His head feels dull, slightly aches, or feels as if bound up tight; wandering pains in his back and limbs; occasional liquid discharges from his bowels; his appetite is variable, and his sleep often disturbed. These symptoms gradually increase for one or two weeks, before the patient finally gives up and takes to his bed.—And when he does so, his *skin* is dry, but only moderatly hot, his pulse is only 85 to a 100, and less forcible than natural; his tongue is covered with a thick dirty white fur, and red along the edges; his intellect is dull and disposed to wander when he is sleeping; all his secretions are diminished, except from the mucous membrane of the ilium, which generally gives rise to from one to six thin evacuations per day; the capillary circulation upon the surface is sluggish; in a word, all the organic actions, are like the mind of the patient, dull and indifferent. Hence it is easy to see how a powerfully sedative dose of 20 grs of Quinine, should subdue the febrile excitement in one class of cases, inducing copious perspiration, and in the other only increase the torpor of the organic actions, and the stupidity of the patient.

